# LncRNA APTR Promotes Uterine Leiomyoma Cell Proliferation by Targeting ERα to Activate the Wnt/β-Catenin Pathway

**DOI:** 10.3389/fonc.2021.536346

**Published:** 2021-03-10

**Authors:** Weiqiang Zhou, Guocheng Wang, Bilan Li, Junjie Qu, Yongli Zhang

**Affiliations:** Department of Obstetrics and Gynecology, Shanghai First Maternity and Infant Hospital, Tongji University School of Medicine, Shanghai, China

**Keywords:** long noncoding RNA (lncRNA) Alu-mediated p21 transcriptional regulator (APTR), uterine leiomyoma, proliferation, ERα, Wnt pathway

## Abstract

The molecular mechanisms by which uterine leiomyoma (UL) cells proliferate are unclear. Long noncoding RNA (lncRNA) is reported to participate in the occurrence and development of gynecological cancers. We investigated the molecular mechanisms that lncRNA uses in UL. We found that lncRNA Alu-mediated p21 transcriptional regulator (APTR) showed higher expression in UL tumor tissues compared with that in normal uterine tissues. APTR induced cell proliferation and colony formation both *in vitro* and *in vivo*. The JASPAR database showed that APTR was likely interacted with ERα, and these molecules were identified *via* laser scanning confocal microscopy and RNA immunoprecipitation analysis. To verify the correlation between APTR and ERα, we overexpressed and underexpressed APTR and simultaneously expressed ERα. The results showed that APTR function was suppressed. APTR increased the expressions of the proteins in the Wnt pathway, and inhibiting ERα eliminated these responses. In conclusion, our data suggest that APTR promoted leiomyoma cell proliferation through the Wnt pathway by targeting ERα, suggesting a new role of APTR in the Wnt signaling pathway in UL.

## Highlights

►APTR induced uterine leiomyoma cell proliferation and colony formation
► APTR directly interacted with ERα and they were co-localized in the nucleus
►APTR promoted leiomyoma cell proliferation through the Wnt pathway by targeting ERα

## Introduction

Uterine leiomyoma (UL) is the most common benign gynecological tumor, with an incidence rate of 30–40% in fertile women (1). UL may lead to asymptomatic complications such as uterine bleeding, severe dysmenorrhea, pelvic pain and infertility and is the leading indication for a hysterectomy (2). However, because the typical marriage age has increased, and many women have not completed childbearing, hysterectomies are often contraindicated (3). Therefore, nonsurgical treatments are urgently needed, and understanding the specific molecular mechanism of UL cell proliferation may improve patients’ quality of life.

*In vitro* and *in vivo* experiments have shown that UL is an estrogen-dependent tumor, and estrogen affects UL promotion and progression (4). Estrogen binds to its receptors and initiates transcription, then promotes UL cell proliferation (5). Estrogen receptors include estrogen receptors (ERs) α and β, and estrogenic action can be influenced by selective estrogen receptors (6). However, whether ERα or ERβ mediate estrogen function and how estrogen affects UL cell proliferation remain unclear.

Aberrant activation of Wnt/β-catenin has been detected in ER-positive breast cancer cells (7). β-Catenin is involved in the canonical Wnt signaling pathway, regulating diverse sets of cellular activities including cell proliferation (8). Enhanced nuclear β-catenin can accelerate interphase by shortening the cell cycle phase and can activate cyclin-D1 and c-Myc (9).

Long noncoding RNA (lncRNA) is reported to participate in gynecological cancers and is associated with the epithelial-mesenchymal transition (EMT) (10). H19 generates miR-675, an EMT-associated gene in prostate cancer (11). LncRNA is also reported to participate in endometrial carcinoma occurrence and development (12).

LncRNA Alu-mediated p21 transcriptional regulator (APTR) is the inhibitor that represses the p21 promoter in human glioblastomas (13). APTR is reported to play a crucial role in osteosarcoma (14), be a potential biomarker for liver cirrhosis (15) and have potential diagnostic value for papillary thyroid cancer (16). In this study, we examined whether APTR plays a pivotal role in UL progression and elucidated the possible mechanism.

## Materials and Methods

### Cells, Patients, and Samples

Three patients were included in the study, and UL tumor tissue and adjacent normal uterine tissue specimens were acquired at the follicular phase. Inclusion criteria for this study were patients who (a) were nonmenopausal, (b) had undergone a hysterectomy because of UL between 12/2016 and 08/2017, (c) were without medical complications, and (d) had no history of hormone therapy. Paraffin-embedded tissues of 34 UL patients were included in the study, and all histopathologic evaluations were performed by experienced pathologists from the Shanghai First Maternity and Infant Hospital.

Ht-UtLM primary cells were collected from 30 patients who had undergone total hysterectomies at the follicular phase and were selected according to the above criteria. All were diagnosed by a pathologist. The detailed steps were previously described (17). Ht-UtLM-1 and the Ht-UtLM-2 came from the same patient’s primary cells. Because the primary cells cannot be subcultured, primary cells from different patients were used for different experiments and there were total 30 patients selected. It was investigated the cells culture was mycoplasma-free.

The Hospital’s Protection of Human Subjects Committee approved the study protocols. Samples were acquired with written informed consent from the Shanghai First Maternity and Infant Hospital affiliated with Tongji University School of Medicine.

### Immunohistochemistry

Immunohistochemical staining was performed using the two-step plus Poly-HRP Anti-IgG Detection System (ZSGB-Bio, Beijing, China) per the manufacturer’s recommendations. Primary antibodies targeting ERα (C-311; cat. no. sc-787, Santa Cruz, CA, USA), ERβ (B-3; cat. no. 373853, Santa Cruz), Ki-67 (cat. no. 373853, Santa Cruz) and β-catenin (12F7; cat. no. sc-59737, Santa Cruz) were used.

### RNA Extraction and Analysis

Total RNA extraction and reverse transcription were performed per the manufacturer’s protocol. Semi-quantitative RT-PCR was performed using the standard protocol from the SYBR Green PCR kit (Toyobo, Osaka, Japan). GAPDH was used as the reference mRNA. DCt values were normalized to GAPDH levels. The 2^–△△Ct^ method was used to measure the gene expression levels. The primer pairs used were as follows: human lncRNA APTR forward: 5′-AGTAGCAGGAGACAGCAT-3′, reverse: 5′-TGACAGCCTTCCACAATC-3′; α-SMA forward: 5′-CTTTCAAGCTGTTCCTGTC-3′, reverse: 5′-TGTGTTTCTCCTCTGTCC-3′; vimentin forward: 5′-GATGGTGTTTGGTCGCATA-3′, reverse: 5′- CGAATGCGCAGCACCAG-3′; KRT-19 forward: 5′-GGCGCCACCATTGAGAA CT-3′, reverse: 5′-GCCAGGCGGGCATTG-3′; and GAPDH forward: 5′-GGCTCCCTTGGGTATATGGT-3′, reverse: 5′-TTGATTTTGGAGGGATCTCG-3′.

### Western Blot Analysis

Western blot analysis was performed to assess protein expression as previously described (18). Primary antibodies included anti-GAPDH (cat. no. G9545) purchased from Sigma, all others were purchased from Santa Cruz, including anti-ERα (sc-8002), anti-KRT-19 (sc-376126), anti-α-SMA (sc-53142), anti-vimentin (sc-6260), anti-actin (sc-8432), anti-c-Myc (sc-373712), anti-β-catenin (sc-7963) and anti-cyclin-D1 (sc-8396).

### Lentiviral Production and Infection

The full-length nucleotide sequence for APTR was obtained from the FLJ cDNA library. It was then inserted at the 3´ end of the APTR in the lentivirus vector (Invitrogen, V49810). The shRNA was constructed as previously described (19).

### Cell Cultures and Colony Formation

Cells were cultured in 10% Dulbecco’s modified Eagle’s medium containing 10% fetal bovine serum (Wisent, Canada) at 37°C with 5% CO2. For the colony-forming assay, cells (6 × 10^2^) were transfected and incubated for 14 days. Colonies (>50 cells) were counted manually and plotted as previously described (18).

### Cell Viability Assay

Cells were plated and grown for 96 h. The number of cells was determined using the 3-(4,5-dimethylthiazol-2yl)-2,5-diphenyltetrazolium bromide (MTT) assay as previously described (20).

### RNA Immunoprecipitation

RIP analysis to assess RNA-binding protein expression was performed as previously described (21). Primary antibodies included mouse monoclonal antibody against ERα (cat. no. sc-787, Santa Cruz) and mouse monoclonal antibody against actin (C-2; cat. no. sc-8432, Santa Cruz).

### Luciferase Assays

Cells were seeded at 1 × 10^5^ cells/well in a 24-well cell plate one day prior to transfection with Superfect according to the manufacturer’s protocol (Tiangen Biotech co LTD, Beijing, China). Luciferase activity was normalized for transfection efficiency using the mutant promoter reporter plasmid, FOPflash (vs. TOPflash, the un-mutated plasmid), as an internal control (22).

### Illumina HiSeq

Total RNA was extracted from cells using TRIzol™ Reagent (#15596026, Invitrogen, Carlsbad, CA, USA) per the manufacturer’s protocol, and ribosomal RNA was removed using the Ribo-Zero rRNA Removal Kit (Epicentre, Madison, WI, USA). Fragmented RNA (average length of ~200 bp) were subjected to first-strand and second-strand cDNA synthesis followed by adaptor ligation and enrichment with a low cycle per the instructions of the NEBNext^®^ Ultra™ RNA Library Prep Kit for Illumina (NEB, USA). The purified library products were evaluated using the Agilent 2200 TapeStation and Qubit^®^ 2.0 (Life Technologies, USA). The libraries were paired-end sequenced (PE150, sequencing reads were 150 bp) using the Illumina HiSeq Xten platform.

Fastp software (v0.17.0) was used to trim adaptor and remove low quality reads to get high quality clean reads. mRNA/LncRNA: hisat2 software (v2.04) was used to align the high quality clean reads to the human reference genome (UCSC hg19). Then, guided by the Ensembl GTF gene annotation file (v75), cuffdiff software (part of cufflinks, v2.2.1) was used to get the gene level FPKM as the expression profiles of mRNA, transcript level FPKM as the expression profiles of LncRNA, and fold change and p-value were calculated based on FPKM, differentially expressed mRNAs/LncRNAs were identified by cuffdiff software. Gene Ontology and KEGG pathway enrichment analysis were performed based on the differentially expressed mRNAs and LncRNA nearby genes. This part of the experiment was performed by Shanghai Yinxi Biomedical Technology Co., Ltd.

### Nude Mouse Study

Ht-UtLM cells (5 × 10^6^) with stably upregulated APTR were subcutaneously implanted into 4- to 6-week-old BALB/c nude mice purchased from Shanghai SLAC Laboratory Animal Company. Tumor growth was measured using a digital caliper every 5 days for 30 days. The mice were sacrificed on day 30 after cell implantation, and the tumor weights were measured.

### Plasmids

The ERαcDNA and ERαRNAi plasmid were purchased from Origene (Maryland, USA) (23).

### Fluorescence In Situ Hybridization

The cDNA encoding APTR was subcloned into the NheI and XhoI sites of pSL-MS2-12x vector (Addgene), named pSL-MS2-APTR, the sequence is as follow: ACTR-F-NheI 5’-TAGCTAGCAGTCCCGCTGACACCTT-3’, ACTR-R-XhoI 5’-ACCTCGAGAACCGTGAGTCCATTAAACCTC -3’. A digoxin (Roche, Mannheim, Germany)-labeled lncRNA-APTR complementary DNA probe was synthesized *in vitro* used for RNA fluorescence *in situ* hybridization (FISH). The following procedures were performed as previously described (24) without slight modification.

### Statistical Analysis

All experiments were repeated in triplicate. Data are expressed as the mean ± SD. Statistical significance between two groups was determined using Student’s t-test. *P*<0.05 was considered statistically significant.

## Results

### Alu-Mediated p21 Transcriptional Regulator Was Overexpressed in Uterine Leiomyoma

**Figure 1:**
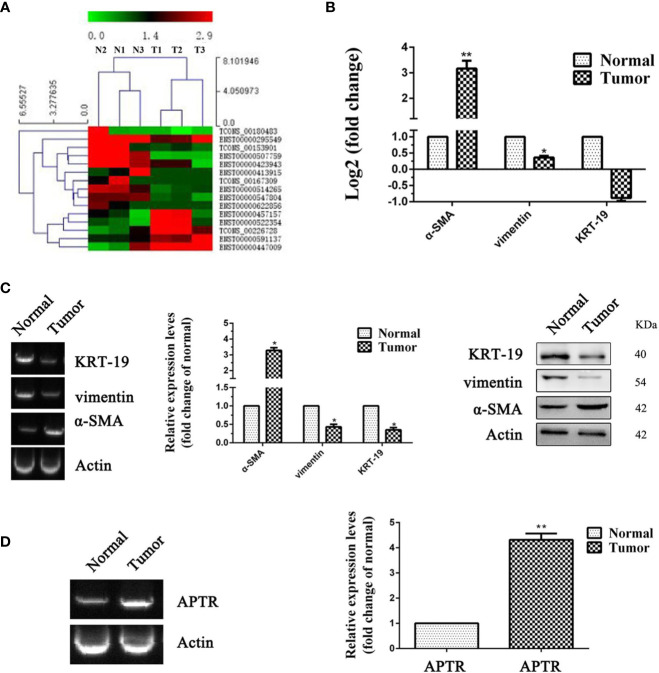
LncRNA expression was high in leiomyoma tumors. **(A, B)** Differences in RNA transcription were detected between normal uterine tissues and leiomyoma tumors. High-level pathway sequencing showed that α-SMA expression was higher in leiomyoma tumors. **(C)** Gel electrophoresis, real-time PCR, and Western blot analyses confirmed higher α-SMA expression and lower vimentin and KRT-19 expression in leiomyoma tumors. **(D)** Gel electrophoresis and real-time PCR analyses confirmed higher APTR expression in leiomyoma tumors. (*P < 0.05, **P < 0.01).

UL tissues and adjacent normal uterine tissues from three patients were used to confirm the APTR expression levels. Illumina HiSeq was used to further verify the higher expression of APTR. APTR expressions in the tissues from the three patients were significantly increased compared with those of the normal tissues (**Figures 1A, B**).

Increased levels of α-SMA and decreased levels of vimentin and KRT-19 identified the leiomyoma tumor cells (**Figure 1C**). Next, real-time PCR analysis confirmed the higher APTR levels in the UL tissues (**Figure 1D**). **Figures 1C, D** showed one representative sample.

### Alu-Mediated p21 Transcriptional Regulator Overexpression Promoted Leiomyoma Cell Proliferation

**Figure 2:**
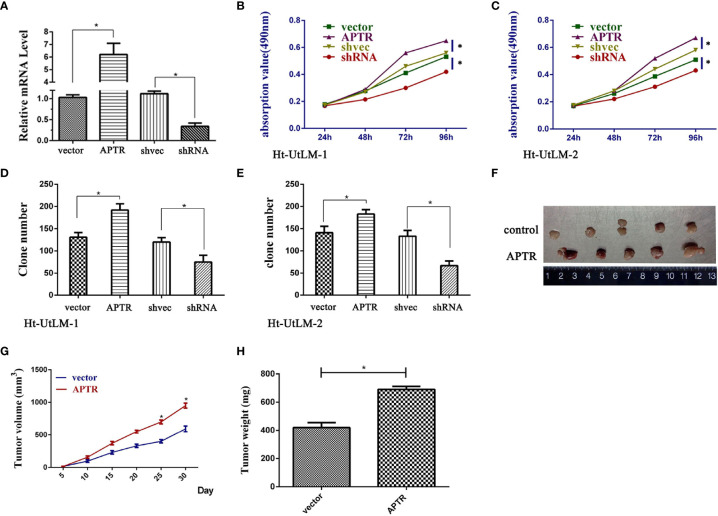
LncRNA APTR induced leiomyoma tumor cell proliferation. Ht-UtLM primary cells derived from leiomyoma tumors were used. **(A)** Stably infected Ht-UtLM cells were generated and identified. Real-time PCR analyses confirmed the efficiency. **(B, C)** Cell proliferation testing was conducted using MTT assays. APTR overexpression increased cell proliferation. Conversely, knocking down APTR decreased cell proliferation. **(D, E)** Colony-formation assays were performed on stably overexpressed or underexpressed APTR for 2 weeks. **(F)** Tumors from xenograft-transplanted nude mice 30 days after subcutaneously injecting APTR-overexpressing or control Ht-UtLM cells. **(G)** Xenograft volumes 30 days after cell injection. **(H)** Xenograft weights 30 days after cell injection.

APTR was overexpressed in UL tumor cells. Thus, we investigated the role of APTR in regulating cell proliferation. We constructed APTR-overexpression and APTR-knockdown lentiviral vectors, infected Ht-UtLM cells, and selected stably infected cell clones for further study. The vectors were compared to a control (**Figure 2A**). MTT analysis showed that APTR**-**infected cells grew much faster than did the controls (**Figures 2B, C**). Colony-formation assays revealed that APTR**-**infected cells formed larger and more numerous colonies than did the controls (**Figures 2D, E**). Conversely, shRNA**-**infected cells had slower proliferation rates and formed smaller and fewer colonies than did the controls.

The stably infected cells were subcutaneously transplanted into BALB/c nude mice. We suspended the cells at 5 × 10^6^ cells/ml, and 100 μl were injected into the flanks of nude mice (n=5). We measured tumor sizes starting 5 days postinjection using the formula, *W^2^* × *L*, every 5 days for 30 days. Mice were then euthanized, and the tumors were excised (**Figure 2F**). Tumor sizes (**Figure 2G**) and weights (**Figure 2H**) from APTR-overexpressing cells were increased compared with the controls.

### Alu-Mediated p21 Transcriptional Regulator Directly Interacted With ERα

**Figure 3:**
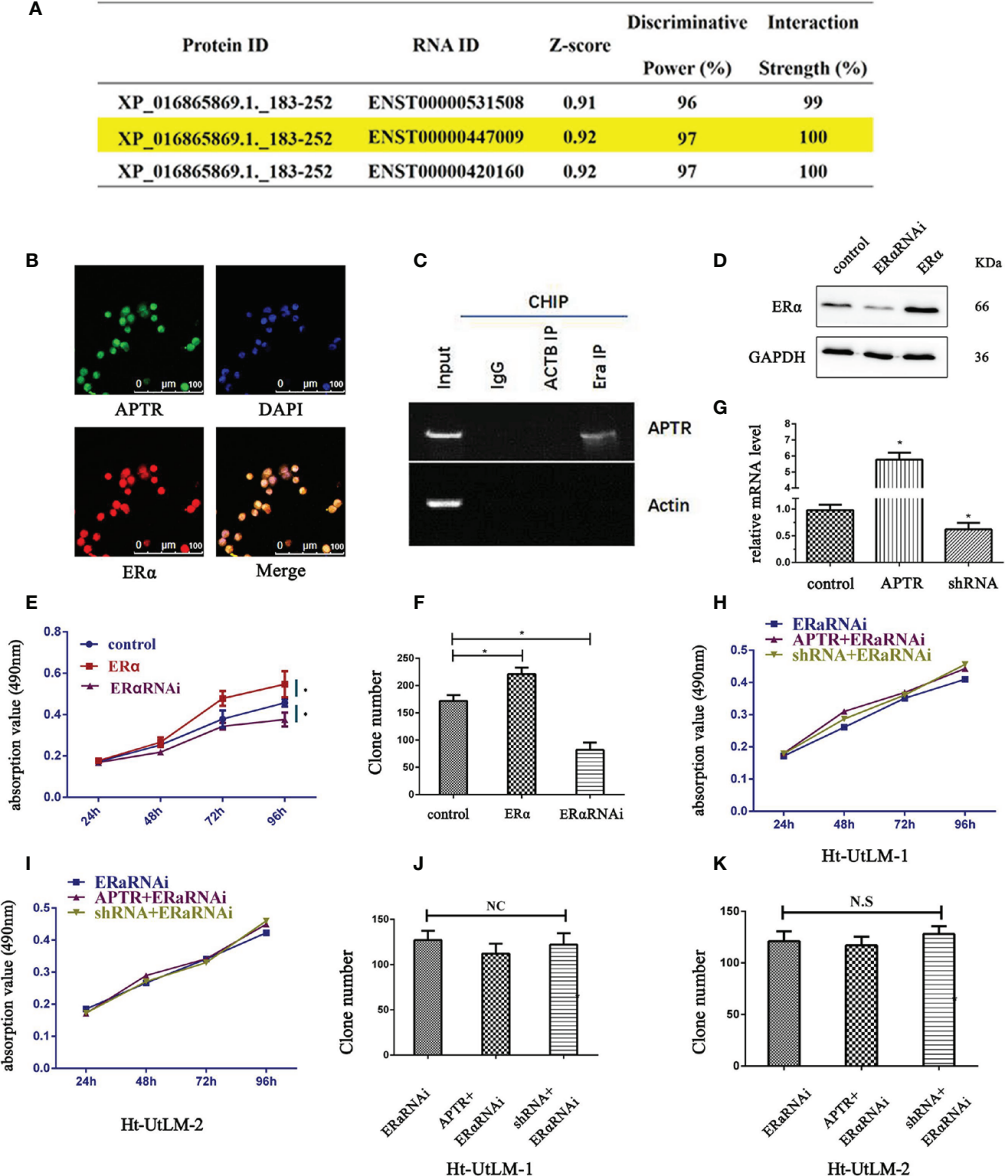
LncRNA APTR induced tumor growth by targeting ERα. LncRNA APTR was likely interacted with ERα. **(A)** LncRNA ENST00000447009 was lncRNA APTR. According to the JASPAR database, APTR was likely interacted with ERα. **(B)** Ht-UtLM cells with elevated APTR expression were seeded in 96-well plates. FISH analysis showed that ERα (red) co-localized with APTR (green) in the nuclei of Ht-UtLM cells. **(C)** RIP showed that APTR was in contact with ERα. **(D)** Ht-UtLM cells were transfected with either ERα cDNA or shRNA plasmids. ERα expression was analyzed by western blot. **(E)** Cell viability was assessed by MTT assay daily for 4 days. **(F)** Colony formation assays were performed on Ht-UtLM cells for 2 weeks. **(G)** Ht-UtLM cells that stably overexpressed or underexpressed APTR were screened *via* real-time PCR. **(H, I)** After overexpressing or underexpressing APTR or ERαRNAi, the cell viability was assessed using MTT assays daily for 4 days. **(J, K)** Colony-formation assays were performed on cells stably overexpressed or underexpressed for 2 weeks. (*P < 0.05).

According to the JASPAR database, lncRNA is likely interacted with ERα (**Figure 3A**). ENST00000447009 is lncRNA APTR, with an interaction strength of 10%. We next explored whether APTR interacts with ERα. The location of APTR was confirmed using FISH assay; APTR and ERα were co-localized in the nucleus (**Figure 3B**). We used RIP to verify the correlation between ERα and APTR, and the results showed that APTR is likely interacted with ERα (**Figure 3C**).

### Alu-Mediated p21 Transcriptional +Regulator Induced Leiomyoma Tumor Cells Proliferation by Targeting ERα

In leiomyoma tumor cells, ERα promoted proliferation, as did APTR. Therefore, the correlation between ERα and APTR were measured. Western blot analysis was used to verify the overexpression and underexpression of ERα (**Figure 3D**). MTT analysis showed that ERα overexpression cells grew much faster than controls (**Figure 3E**). Colony formation assays also revealed that ERα overexpression cells formed larger and more colonies than controls (**Figure 3F**). Conversely, ERα α cells had slower proliferation rates and formed smaller and fewer colonies than controls.

APTR levels were overexpressed and underexpressed (**Figure 3G**). The effect of APTR on growth was abolished when simultaneously infected with ERαRNAi (**Figures 3H, I**). Colony-formation assays revealed the same results. Colonies formed by the APTR- and ERαRNAi-infected cells did not significantly differ from those formed by the control cells (**Figures 3J, K**).

### APTR Promoted Leiomyoma Cell Proliferation Through the Wnt Pathway by Targeting ERα

**Figure 4:**
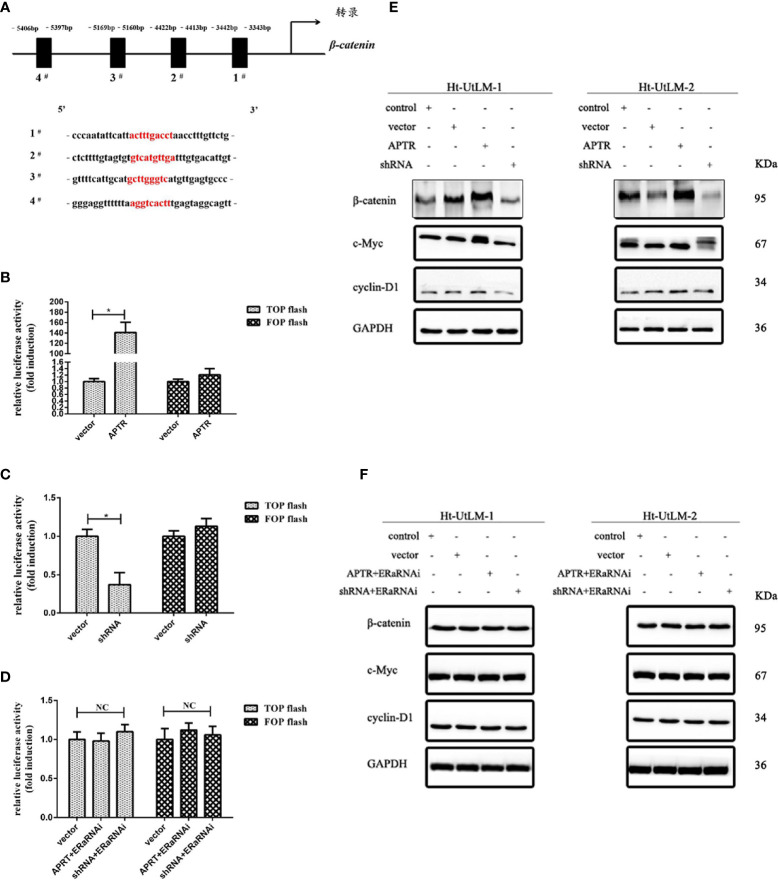
APTR activated the canonical Wnt pathway in Ht-UtLM cells by targeting ERα. **(A)** β-Catenin promoter has four ERα binding sites. **(B)** Cells were infected with or without APTR together with the TCF-luciferase reporter (TOPflash) or mutant TCF-luciferase reporter (FOPflash) for 24 h. Upregulated APTR increased TOPflash activation in Ht-UtLM cells. **(C)** Downregulated APTR repressed TOPflash activation in Ht-UtLM cells. **(D)** Infection with APTR and ERαRNAi did not affect the basal TOPflash activity. **(E, F)** Ht-UtLM cells were infected with APTR or shRNA alone or together with the ERαRNAi. Western blot analysis was performed with GAPDH as the loading control. (*P < 0.05, **P < 0.01).

By bioinformatic analysis, β-catenin promoter has four ERα binding sites (**Figure 4A**). β-catenin promoter luciferase reporter (TOPflash) was used to investigate the regulation of β-catenin by APTR. The Ht-UtLM cells were coinfected with APTR or shRNA and TOPflash, and the activity was examined. The results showed that TOPflash activity was increased by cotransfection with APTR and decreased by shRNA (**Figures 4B, C**). When the cells were cotransfected with both APTR and ERαRNAi or shRNA and ERαRNAi, both cell groups lost their responses (**Figure 4D**).

Western blotting was used to further validate the effect of APTR. APTR upregulated β-catenin expression and simultaneously indirectly upregulated c-Myc and cyclin-D1, downstream proteins of the Wnt signaling pathway. Conversely, the β-catenin and downstream cyclin-D1 and c-Myc expression levels did not differ from those of the control groups. When the cells were coinfected with both APTR and ERαRNAi or shRNA and ERαRNAi, the changes in protein expressions between the two groups did not significantly differ (**Figures 4E, F**). These results indicated that APTR activated the Wnt pathway by targeting ERα.

## Discussion

In this study, we identified a new effect of lncRNA APTR, which targets the p21 promoter. We found high APTR expression levels in the leiomyoma tumor tissues using Illumina HiSeq and real-time PCR experiments to examine leiomyoma tumor tissues and adjacent normal cells. Bioinformatics analyses showed that APTR is likely interacted with ERα. Knocking down the ERα level abolished the effect of APTR on UL cell proliferation. This effect on APTR reveals a new role for ERα in the Wnt pathway and represents the first identification of the lncRNA and ERα pathway.

APTR represses the CDKN1A/P21 promoter, which has been correlated with cell proliferation in several cancers (13, 15). Downregulation of APTR has been correlated with tumorigenesis in papillary thyroid cancer and anaplastic thyroid cancer (16). In cirrhotic patients with portal hypertension, APTR was considered a prognostic marker, and higher APTR expression was associated with a poor prognosis (25).

Given the recently identified role of APTR, we first detected APTR expression in leiomyoma tissues and adjacent normal tissues. As expected, APTR had a higher expression in leiomyoma tissues. Further investigation of APTR’s function would be of interest since APTR overexpression promoted proliferation of the UL cell line, but underexpression of APTR had the opposite effect. Until now, the mechanism of APTR regulation as it relates to UL cell proliferation was unclear.

Long noncoding RNA (lncRNA) with more than 200 nucleotides is considered a transcript and is not translated into proteins (26). With little primary sequence conservation, many lncRNAs are localized to the nucleus (27), while some can encode small proteins (28). Bioinformatics analysis revealed that APTR was likely interacted with ERα, and we verified this using RIP. To further assess the function, we overexpressed APTR and underexpressed ERα simultaneously, which inhibited the effect of APTR. Underexpressing APTR and ERα simultaneously also inhibited the effect.

UL is reported to be estrogen-dependent (29), and estrogen that was uptaken or exposed bound to the estrogen receptor by acting with an estrogen-like effect (4). Estrogen can overexpress ERα and upregulate IGF-1 expression (30) and the VEGF pathways (31). The ERα signaling pathway can crosstalk with the TGF-β signaling pathway and mediate estrogen to promote UL cell proliferation (32).

Variant estrogen receptors have also been reported, such as ERα36, which is located in the mitochondria in Ht-UtLM cells. These receptors are associated with mitochondrial proteins and considered to be nongenomic signals with pivotal functions. Bisphenol A induced UL cell proliferation through ERα36 as a nongenomic signaling pathway (33).

ERβ was also found in UL. ERα expression was shown to be higher than that of ERβ (34). In our study, specimens were taken at the follicular phase, and ERβ had low expression in leiomyoma tumor tissues (**Supplemental Figure 1**). Furthermore, the effect on the UL cell proliferation was related to ERα.

Interestingly, in this study, the effect of APTR on UL cell proliferation was offset by ERα. Studies have reported that different APTR levels affect the cellular biological activity; for example, knocking down APTR inhibited TGF-β1-induced upregulation of α-SMA in hepatic stellate cells (15).

Aberrant activated Wnt/β-catenin was detected in this study. We also detected β-catenin activity in ER-positive UL patients, which verified the effect of APTR in the Wnt/β-catenin pathway. Knocking down ERα depleted this effect. We also found a new checkpoint for ERα. LncRNA APTR promotes UL cell proliferation by activating the Wnt/β-catenin pathway, and ERα was the target of the effect of APTR.

Current studies are done in primary cells from only one patient for one and the same experiment, to avoid patient-specific issues, results will be confirmed in more patients in the near future.

## Data Availability Statement

The original contributions presented in the study are publicly available. This data can be found here: [https://www.ncbi.nlm.nih.gov/geo/query/acc.cgi?acc=GSE168497/GSE168497].

## Ethics Statement

The studies involving human participants were reviewed and approved by The Hospital’s Protection of Human Subjects Committee. The patients/participants provided their written informed consent to participate in this study. The animal study and study protocols were reviewed and approved by The Hospital’s Protection of Human Subjects Committee. Written informed consent was obtained from the individual(s) for the publication of any potentially identifiable images or data included in this article.

## Author Contributions

All authors contributed equally to this paper. All authors contributed to the article and approved the submitted version.

## Conflict of Interests

The authors declare that the research was conducted in the absence of any commercial or financial relationships that could be construed as a potential conflict of interest.

## Supplementary Material

Supplementary Figure 1 IHC staining shows ERα and ERβ expressions in uterine leiomyoma tumor compared to adjacent normal uterine tissues. The expressions of ERα and ERβ in uterine leiomyoma tumor and adjacent normal uterine tissues. The scale bar is 100 μm.
