# Supplementary figures and images for: LncRNA APTR Promotes Uterine Leiomyoma Cell Proliferation by Targeting ERα to Activate the Wnt/β-Catenin Pathway
Source: Front Oncol. 2021 Mar 10;11:536346. doi: 10.3389/fonc.2021.536346 (PMC7989393; doi:10.3389/fonc.2021.536346)

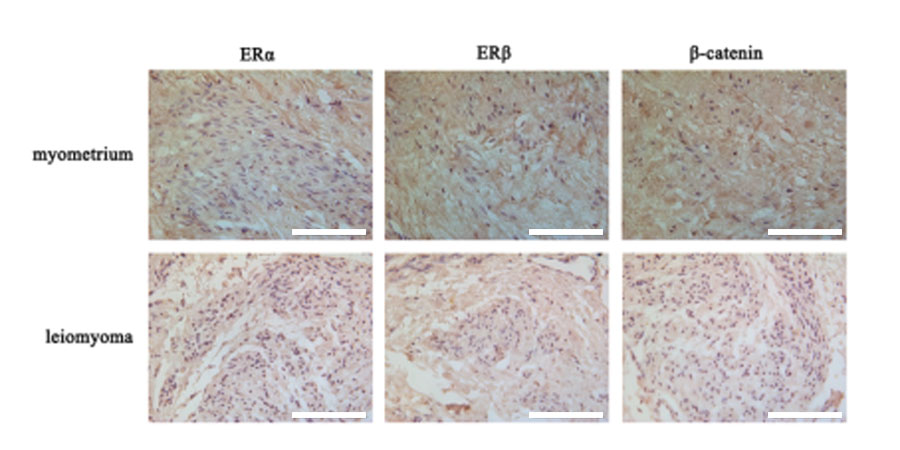

Supplement: Supplementary Figure 1 — IHC staining shows ERα and ERβ expressions in uterine leiomyoma tumor compared to adjacent normal uterine tissues. The expressions of ERα and ERβ in uterine leiomyoma tumor and adjacent normal uterine tissues. The scale bar is 100 μm. [file Image_1.jpg]
